# Fear of falling and associated activity restriction in older people. results of a cross-sectional study conducted in a Belgian town

**DOI:** 10.1186/0778-7367-70-1

**Published:** 2012-01-03

**Authors:** Elise Mendes da Costa, Thierry Pepersack, Isabelle Godin, Martine Bantuelle, Bernard Petit, Alain Levêque

**Affiliations:** 1 Fonds de la Recherche Scientifique - FNRS (research fellow), Department of Epidemiology and Health Promotion, School of Public Health, Université Libre de Bruxelles (U.L.B.), Route de Lennik, 808 - CP 596; B-1070 Brussels, Belgium; 2 Geriatrics, Erasme University Hospital, Université Libre de Bruxelles (U.L.B.), Route de Lennik, 808; B-1070 Brussels, Belgium; 3 Department of Epidemiology and Health Promotion, School of Public Health, Université Libre de Bruxelles (U.L.B.), Route de Lennik, 808 - CP 596; B-1070 Brussels, Belgium; 4 Educa Santé, non-profit organisation, Avenue Général Michel, 1b; B-6000 Charleroi, Belgium

**Keywords:** Accidental falls, activity restriction, aged, aged 80 and over, fear of falling

## Abstract

**Objectives:**

This article aims at describing, in a Belgian town, the frequency of the fear of falling and of subsequent activity restriction among non-institutionalised people aged 65 years and over, and at identifying persons affected by these two issues.

**Methods:**

Cross-sectional survey conducted in Fontaine l'Evêque (Belgium) in 2006, using a self-administered questionnaire.

**Results:**

The participants could fill in the questionnaire on their own or with the help of a third party if needed. The latter were not taken into account in this article. Analyses covered 419 questionnaires. Fear of falling and activity restriction were reported by, respectively, 59.1% and 33.2% of participants. They were more frequent among fallers but also affected non-fallers. In logistic regression analyses: gender, the fact of living alone and the number of falls were significantly associated with fear of falling; gender, age and the number of falls were significantly associated with activity restriction.

**Conclusions:**

Our study, despite various limitations, shows the importance of fear of falling and of subsequent activity restriction among older people, among fallers as well as among non-fallers. It also provides information, though limited, concerning persons affected by these two issues in Belgium, and in other contexts as well. Given the ageing of our populations, it is important to take these problems into account when caring for older people.

## Introduction

Older people currently represent a significant proportion of European [1] and Belgian [2] populations. According to various prospective studies reviewed by Rubenstein and Josephson [3], 30 to 60% of community-dwelling older people sustain a fall each year, about half of them falling several times. Besides the important physical repercussions that falls can sometimes have [3], it seems that they may also cause psychological difficulties for many older people [4]. Among these difficulties, we notably find fear of falling and activity avoidance [4].

According to the results of several studies, one can estimate that between 20.8 and 57% [5-10] of non-institutionalised people aged 62 years and over feel this fear. In two different studies, activity restriction affects, respectively, 37.9 [10] and 43% [5] of non-institutionalised older people and between 44 and 56% of people who are afraid of falling [5,8,9]. It is important to emphasise that, as mentioned in the literature, fear of falling and activity restriction do not only affect fallers [10,11]. Fear of falling is reported for example by between 12 and 65% of community-dwelling people aged 60 years and over who have never sustained a fall [12].

The link between fear of falling and falls seems to go both ways: fear of falling is more frequent amongst fallers and people feeling this fear are more at risk of falling [8]. This increase in the risk of falling would be linked, according to some authors, to the activity restriction brought about by this fear [5,8,13,14], a restriction that may cause, amongst other things, muscle atrophy [14], deconditioning [5,13,14] and worse balance [14]. And, this in turn could feed the fear and avoidance [15]. Fear of falling and activity avoidance seem moreover to play a role in the "transition to physical frailty" [15].

To our knowledge, few data are available for Belgium on the frequency of fear of falling and of subsequent activity restriction, and on the characteristics of the persons affected by these problems. The data of Delbaere et al. [15,16] provide some information concerning the fear of falling and the fear-related avoidance of activities (measured in their study using a Dutch version of the modified Survey of Activities and Fear of Falling in the Elderly scale). In their study, fear of falling was reported by 56.4% of community-dwelling people aged 60 years or over [16]. They also notably examined the associations between fear-related avoidance of activities and physical performance, postural control, muscle strength, and the fall history [15].

This paper aims at describing the frequency of the fear of falling and subsequent activity restriction in one Belgian town, and at identifying the people affected by these two issues in this town.

## Methods

### Survey

The results presented here come from the secondary analysis of a cross-sectional survey, whose general objectives were to describe: the frequency, circumstances and consequences of falls, the frequency and circumstances of fear of falling and of related activity restriction, and the modifications made to the home environment or the ones that would be necessary to decrease the risk of falling. This survey was conducted between April and August 2006 in the semi-rural town of Fontaine l'Evêque (FLE), a town of 16687 inhabitants (on 1^st ^January 2006)^1 ^located in the Walloon region of Belgium. FLE has been developing a "safe community" [17] approach. "By a Safe Community is meant a local community - often a municipality - where there is an active injury-prevention programme covering all ages, environments and situations, and also where networks of public authorities, health services, voluntary organisations, enterprises and interested individuals work together" ([17], page 49). These "Safe Communities" have to fulfil a certain number of criteria, including implementing "Programs that document the frequency and causes of injuries" ([17], page 101). The survey was aimed at all persons aged 65 years and over living at home in this town. The local administration sent the self-administered questionnaires to the persons identified as eligible, asking them to send them back after completion. A reminder was published in the local press, to increase the participation rate.

The participants in the survey could fill in the questionnaire on their own or with help. We decided, in the analyses presented here, not to take into account the people who had filled in their questionnaires with help. This decision was made because of the observation reported by Higashi et al. [18] that proxies tend to be more worried about falls than older people themselves. Moreover, differences were observed in our sample between the persons who filled in the questionnaires with help and those who did it alone. In the latter, the proportion of women, of persons aged 75 years and over and of people living alone, and the frequency of fear of falling and of activity restriction were lower. Some differences also existed between the two groups in the associations between the independent variables and the two studied outcomes. The persons taken into account in our analyses are named 'the participants' in the rest of the text.

### Data collection

Different types of information were gathered; only the ones taken into account for this article are presented here in detail.

#### Independent variables

Various demographic data were collected (gender, date of birth, living alone or not). Three age categories were set: a) 65 to 74, b) 75 to 84, c) 85 years and over. Based on two questions regarding fall history, we constructed a variable 'number of falls within the past 12 months: 0 - 1 - 2 or more'.

Other variables seem to be associated with fear of falling and/or with activity restriction [19]. The data used in this article come from an existing study, of which the objectives were not only to examine the risk factors of fear of falling and of activity restriction. Therefore, we were limited by the type of information collected and also, for some variables, by the number of subjects.

#### Outcomes

The fear of falling was assessed through the question "Are you afraid of falling? Yes - No". The activity restriction due to fear of falling was researched through the question "Have you stopped or do you less frequently carry out some of your activities due to fear of falling? Yes - No"; all participants were asked this last question, whether they were afraid of falling or not.

### Statistical methodology

Usual descriptive statistics (here, frequencies) were used to describe the sample. To see if the subjects taken into account in our analyses were comparable to the FLE, Walloon and Belgian populations aged 65 years old and over living at home (named FLE, Walloon and Belgian populations in the rest of the text), we compared their characteristics with those of these populations^2 ^using the Chi-square. To study the associations between the independent variables and both outcomes, we used the Chi-square and the Chi-square for trend when applicable. To measure the force of these associations, the raw Odds Ratios (ORr) and their 95% confidence intervals (95% CI) were computed; the exact limits of the 95% CI were computed whenever Cornfield limits were not precise enough. Logistic regression models were constructed. Their aim was not to predict the studied outcomes as good as possible, but, given that the information we had on the associated factors was limited, to simultaneously take into account the effect of the different variables. We forced the entry of all independent variables in the two models. Model fit was checked by the application of the Hosmer and Lemeshow's goodness-of-fit test and by residual analysis. The adjusted OR (ORa) and their 95% CI were derived from the models and are presented in the tables, together with the p-value of Wald's Chi-squares. The analyses were done with SPSS 16.0 and EpiInfo 3.3.2 software. The level of significance of 0.05 was chosen for all analyses.

## Results

### Participation in the whole survey (Figure 1)

Out of the 2812 questionnaires sent, 514 were sent back (participation rate: 18.3%). Thirteen questionnaires were excluded (- incalculable age (n = 6) or age under 65 years (n = 1) - institutionalised people (n = 2) - people unable to walk (n = 1) or in wheelchairs (n = 2) - people unable to fill in the questionnaire (n = 1)). Out of the 501 remaining questionnaires, 82 had been filled in with the help of a third party. The analyses presented here therefore covered 419 questionnaires.

**Figure 1:**
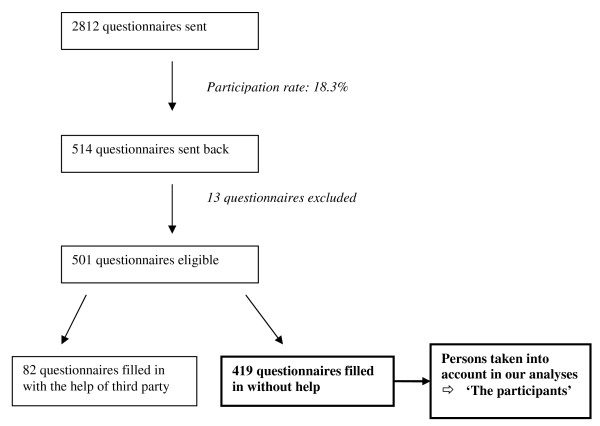
**Flow-chart of recruitment** [FLE survey, 2006].

### Characteristics of participants

Characteristics of participants are presented in Table 1, along with those of FLE, Walloon and Belgian populations. Despite some small non-significant differences, our sample is globally comparable to those three populations. One exception is that there were significantly more people living alone in our sample than in the Belgian population.

**Table 1:** Characteristics of participants and of Fontaine l'Evêque, Walloon and Belgian populations aged 65 years old and over living at home [FLE survey, 2006]

	Participants (total n = 419)		FLE population, aged 65 years and over living at home (2006)^a^			Walloon population, aged 65 years and over living at home (2006)^b^			Belgian population, aged 65 years and over living at home (2006)^c^		
	**n**	**%**	**n**	**%**	**p^d^**	**n**	**%**	**p^e^**	**n**	**%**	**p^f^**

**Gender**	416		2 756		0.119	539 234		0.590	1 712 256		0.867
Female		57.7		61.7			59.0			57.3	
**Age (in years)**	419		2 756		0.719	539 234		0.742	1 712 256		0.359
65 - 74		51.3		51.5			53.1			54.7	
75 - 84		40.6		41.5			38.8			37.3	
85 and over		8.1		7.0			8.1			8.0	
**Does the person live alone?**	415		2 756		0.866	539 234		0.277	1 712 256		0.039
Yes		36.4		36.0			33.9			31.7	
**Number of falls within the past 12 months**	414			DU			DU			DU	
0		68.4									
1		12.3									
2 or more		19.3									

^a, b, c ^Data on 1st January 2006 (data provided by the *FPS Economy, S.M.E.s, Self-employed and Energy - Statistics Belgium*).

^d, e, f ^Comparison between the characteristics of the participants and those of FLE, Walloon and Belgian populations aged 65 years old and over living at home.

DU: data unavailable

### Fear of falling and activity restriction

Among the participants, 59.1% (234/396) said that they were afraid of falling, and 33.2% (126/380) reported having reduced or stopped some of their activities due to fear of falling. Activity restriction was reported by 51.8% (113/218) of the people who were afraid of falling, and by 6.4% (10/156) of those who said they were not afraid.

### Factors associated with the fear of falling (Table 2)

In our bivariate analysis, fear of falling was significantly more frequent among women and among people living alone. The proportion of people who were afraid of falling also increased significantly with age and with the number of falls during the last year. It must be noted that 50.2% of the persons who did not fall feared falling.

**Table 2:** Relationship between the fear of falling and gender, age, the fact of living alone and the number of falls: bivariate analysis and logistic regression [FLE survey, 2006]

	Bivariate analysis				Logistic regression (n = 390)	
	**n**	**% of people being afraid of falling**	**ORr (95% CI)^a^**	**p**	**ORa (95% CI)^b^**	**p**

**Gender**						
Female	227	68.3	2.40 (1.55 - 3.70)		1.98 (1.27 - 3.08)	
Male	167	47.3	1	< 0.001	1	0.003
**Age (in years)**						
85 and over	32	78.1	3.11 (1.23 - 8.87)^c^		2.13 (0.78 - 5.82)	
75 - 84	162	62.3	1.44 (0.93 - 2.25)		1.22 (0.77 - 1.94)	
65 - 74	202	53.5	1	0.005^d^	1	0.290
**Does the person live alone?**						
Yes	142	76.1	3.18 (1.96 - 5.16)		2.15 (1.31 - 3.54)	
No	252	50.0	1	< 0.001	1	0.003
**Number of falls within the past 12 months**						
2 or more	73	82.2	4.58 (2.32 - 9.21)		3.45 (1.76 - 6.76)	
1	45	73.3	2.73 (1.29 - 5.86)		2.36 (1.34 - 4.91)	
0	275	50.2	1	< 0.001^d^	1	< 0.001

^a ^ORr (95% CI) = raw Odds Ratios (95% Confidence Intervals)

^b ^ORa (95% CI) = adjusted Odds Ratios (95% Confidence Intervals)

^c ^Exact limits

^d ^Chi-square for trend

In the logistic regression model, the variables that were significantly associated with the fear of falling were gender, the fact of living alone and the number of falls.

### Factors associated with activity restriction (Table 3)

In our bivariate analysis, activity restriction was significantly more frequent among women and among people living alone. The frequency of this also increased significantly with age and with the number of falls within the past 12 months, affecting however one quarter of the subjects who did not fall.

**Table 3:** Relationship between activity restriction and gender, age, the fact of living alone and the number of falls: bivariate analysis and logistic regression [FLE survey, 2006]

	Bivariate analysis				Logistic regression (n = 374)	
	**n**	**% of people having restricted their activities due to fear of falling**	**ORr (95% CI)^a^**	**p**	**ORa (95% CI)^b^**	**p**

**Gender**						
Female	212	40.6	2.15 (1.34 - 3.46)		1.92 (1.18 - 3.14)	
Male	166	24.1	1	0.001	1	0.009
**Age (in years)**						
85 and over	31	58.1	3.90 (1.67 - 9.18)		2.83 (1.19 - 6.69)	
75 - 84	158	36.7	1.64 (1.01 - 2.65)		1.44 (0.88 - 2.34)	
65 - 74	191	26.2	1	< 0.001^c^	1	0.046
**Does the person live alone?**						
Yes	140	45.7	2.39 (1.50 - 3.81)		1.56 (0.95 - 2.55)	
No	238	26.1	1	< 0.001	1	0.077
**Number of falls within the past 12 months**						
2 or more	69	58.0	3.96 (2.20 - 7.13)		3.04 (1.70 - 5.42)	
1	45	35.6	1.58 (0.77 - 3.24)		1.33 (0.66 - 2.68)	
0	263	25.9	1	< 0.001^c^	1	0.001

^a ^ORr (95% CI) = raw Odds Ratios (95% Confidence Intervals)

^b ^ORa (95% CI) = adjusted Odds Ratios (95% Confidence Intervals)

^c ^Chi-square for trend

In the logistic regression model, these associations remained significant, except the association with the fact of living alone.

## Discussion

Fear of falling was frequent in our study, affecting nearly 60% of participants. Our results are comparable, though sometimes slightly higher, to those of previous studies [5,7,10,16], including one led in Belgium [16]. Nevertheless, they are higher than those of other authors [6,8,9,20]. Activity restriction due to fear of falling was also frequent, affecting one third of the participants. This frequency is close to that observed in a Dutch survey in which 37.9% of people aged 70 and over avoided some activities due to fear of falling [10]. It is however lower than that of another survey in which 43% of participants aged 62 years and over living in public senior housing developments reported that they were not doing some activities or had stopped these because of fear of falling [5]. It is also lower than in another study in which 41.2% of people aged 65 years and over were limiting going outdoors because of fear of falling, but this was led among persons receiving home care services [21]. In our study, 51.8% of people afraid of falling reported a restriction of activities. This is in agreement with other studies in which activity restriction affects between 44% and 56% of people who are afraid of falling [5,8,9]. It should be noted that in our study, among the people who said they were not afraid of falling, 6.4% (= 10 persons) nonetheless reported a reduction in some of their activities due to fear of falling. In the study of Howland et al., among people who were not afraid of falling, 27% reported a restriction of some activities due to fear of falling [5]. According to these authors, one hypothesis is that the restriction of people's activities has allowed them to cope with this fear, leading them to no longer being afraid of falling [5]. Our data did not allow us to test this hypothesis. We nevertheless kept these persons in our analyses.

In our study, in logistic regression analysis, factors associated with the fear of falling were being a woman, living alone and the number of falls within the past 12 months. Hereafter, we compare our logistic regression observations with the multivariable results of other studies [5,6,8,10,11,20,22,23] that have also examined the factors associated with the fear of falling, among which two studies that were conducted among women only [11,23], and one among Mexican-American older people [22]. Our observation of a higher risk among women was also seen in other cross-sectional studies [5,10,20] and in two longitudinal studies, in which women developed this fear more frequently [6,22]. The link between the fact of living alone and the fear of falling was also observed in two other studies [11,23], one of which also showed that living alone was associated with the persistence of this fear [23]. This association between the fact of living alone and the fear of falling was however not observed by other authors [5,10]. The association between the number of falls and the fear of falling is in agreement with the literature in which different variables linked to the fact of having sustained a fall are associated with the fear of falling [5,10,11,23], its development [6,8,22] or persistence [23]. Nevertheless, in some studies [5,23], associations vary according to the variables used in relation to falls, and Filiatrault et al. [20] did not find a significant association between the fall history and the fear of falling. The absence of association, in our study in logistic regression analysis, between age and the fear of falling is consistent with other studies that did not find links between age and the fear of falling [5,11,20,23], its development [6,22,23] or persistence [23]. Zijlstra et al. [10] observed however an association between the increase in age and the fear of falling.

Concerning activity restriction, the variables that were associated with it in logistic regression analysis were: gender, age, and the number of falls. Hereafter, we compare our logistic regression observations with the multivariable results of other studies that have examined the factors associated with this activity restriction [5,9,10,21], including two studies on people who said they were afraid of falling [5,9] and one among people aged 65 years and over receiving home care services [21]. Other authors also observed a higher risk of activity reduction among women [10,21] and among older people [10]; these associations are however not found in certain studies [5,9]. The links we observe between the number of falls and activity restriction are in agreement with the association with the fall history observed in other studies [9,10,21]; variables linked to the fall history used in these studies [9,10,21] are however different from the variable we used (recall period - severity of the falls - number of falls). Moreover, in the study of Murphy et al. [9], association between activity restriction and fall history differs according to the variable used and other authors did not observe significant links between two variables related to the fact of having fallen and activity restriction [5]. The fact that in logistic regression analysis living alone was not associated with activity restriction is in agreement with other studies [5,9,10].

An important point to emphasise is that, as observed by other authors [10,24], both the fear of falling and activity restriction also often affect people who have not fallen (in our study, respectively 50.2% and 25.9%).

The cross-sectional survey on the problem of falls, from which our data originate, was carried out in FLE within the framework of a fall prevention programme among older people. This programme was developed within the broader framework of the development of a "safe community" [17] approach by this town. The "Safe Communities" are communities "(...) where there is an active injury-prevention programme covering all ages, environments and situations (...)" ([17], page 49) and it is recommended that these communities implement "Programs that document the frequency and causes of injuries" ([17], page 101). The cross-sectional survey was conducted notably to respond to this recommendation. In the fall prevention programme, different actions were undertaken. A 'fall prevention day' was organised for the older people of the town. During this day, the results of the cross-sectional survey were presented; these results were very interesting for these older persons because they were local and thus concerned them directly. Other actions included the organisation of a 'balance workshop' for older people and the creation of a 'card of connection', whose aim was to enable the professionals who care for the older persons to communicate on the health status of the patients in the framework of fall prevention.

Our survey presents various limitations. First, its cross-sectional nature does not allow conclusions of causality on the observed associations.

Next, our participation rate is rather low. This may, as suggested by Arfken et al. [25], represent a bias and lead to an underestimation of the studied issues, since people that are more afraid of falling may answer less frequently than others [25]. Since we have no information on the non-respondents, no conclusion can be drawn on the implications of this response rate. Nevertheless, our sample is globally comparable to the FLE, Walloon and Belgian populations, except for the number of people living alone, which was higher in our study than in the Belgian population, which would lead to an overestimation of the frequency of the fear of falling. However, information concerning the characteristics of these populations are limited; we thus cannot exclude other differences, concerning for example their health or functional status.

Another limitation may lie in the decision to only include respondents who had filled in the questionnaire alone. These persons may be healthier than those who needed help to fill in their questionnaires. In comparison with the latter, they notably mentioned being afraid of falling or restricting their activities less frequently; they are also younger and among them, the proportions of women and of people living alone are lower. This could lead to an underestimation of the frequency of the studied issues, and also limits the generalisability of our results.

According to the literature, other factors seem associated with fear of falling and/or with activity restriction [19]. Information available on risk factors in our survey was limited, which limits our study to some extent.

Another limitation is linked to the absence of information on the cognitive status of participants, which some authors took into account as an exclusion criteria [8,25].

Yet another limitation could also lie in the way we measured the fear of falling and activity restriction. To our knowledge, no data is available on the psychometric properties of the measures that we used when formulated in French; neither did we find a study of their use in the Belgian context. Nevertheless, according to the literature, if the evidence of the reliability of the question "Are you afraid of falling?" seems "adequate", the evidence of its responsiveness is "weak" and there is no data as to its validity [4]. As to the question "Has fear of falling made you avoid any activities?", the evidence of its reliability seems "weak" and there is no data as to its validity and responsiveness [4]. If these data relate to questions in English, and if the wording of the question on activity restriction is slightly different from ours, this would also suggest some limits. Moreover, there are discussions in the literature as to the character of the words "fear" or "afraid", which may be too "loaded" (in quotation marks in the authors' text) to describe what people feel with regards to falling [4]. Another limitation could lie in the fact that the question on activity restriction was asked in a general way and not in relation with particular activities (basic activities of daily living,...).

Finally, other "fall-related psychological instruments" exist, among which the Falls Efficacy Scale, which is, as noted by Moore and Ellis, "the most widely used" and "Commonly referred to as the 'gold standard'" [26]; not having used this may also limit our study.

Despite its limitations, our study is interesting. It shows again, in general and specifically for Belgium, the frequency of the fear of falling and of related activity restriction among older people. It also provides information, though limited, concerning people affected by these issues in a Belgian town. As mentioned above, data concerning these issues and the persons that they affect are scarce in Belgium, which shows the added value of our study and its interest in the Belgian context. Another strength of our survey is the use of a self-administered questionnaire that may, according to some authors [7], be interesting, given the reluctance of some older people to talk about their fear [7] - it may however also be an explanation for our low participation rate and raises questions on the validity of the information we gathered.

As Tinetti and Powell said in 1993, "Neither falling nor fear of falling should be considered inevitable accompaniments of aging. Rather, they are specific entities, with specific risk factors which may be amenable to intervention" [27]; and as a recent study shows, some existing interventions, such as community-based tai chi or home-based exercise interventions, may reduce the fear of falling [28]. Besides, it is suggested to take into account the multifactoriality of fear of falling when proposing interventions aimed at decreasing fear of falling [12]. It seems fundamental to us that healthcare providers who are in touch with older people are made aware of these issues and of the persons affected by them, and that they can integrate the detection, prevention and treatment of these problems in their work. Moreover, Murphy et al. [29] suggest that it may be interesting that healthcare providers start to talk about the fear of falling and its consequences, since older people do not necessarily talk about this fear or their falls.

More research and more efforts are still needed: studies that evaluate the interventions that may reduce the fear of falling would be necessary [28] and, according to some [29], many interventions that can reduce the fear of falling are not part of fall prevention programmes. Concerning Belgium more specifically, if our data and these of Delbaere et al. [15,16] provide some information about fear of falling and related activity restriction, it would be interesting to study these issues and their risk factors more extensively.

## Conclusion

Despite its various limitations, our study shows the frequency of fear of falling and of related activity restriction among older persons, among fallers as well as among non-fallers. It also provides information, though limited, concerning persons affected by these issues in Belgium. Given the current and the expected ageing of our populations, it is important to take into account these problems when caring for older people.

## Competing interests

The authors declare that they have no competing interests.

## Authors' contributions

EM participated in the design of the study, performed the data entry and the statistical analysis, and drafted the manuscript. TP, IG and BP critically reviewed the text. MB participated in the design of the study, coordinated the data collection and critically reviewed the text. AL participated in the design of the study and in the redaction of the manuscript. All authors read and approved the final manuscript.

## Endnotes

^1, 2 ^Data provided by the *FPS Economy, S.M.E.s, Self-employed and Energy - Statistics Belgium*.
